# Hospitalisation costs of metastatic melanoma in France; the MELISSA study (MELanoma In hoSpital coSts Assessment)

**DOI:** 10.1186/s12913-017-2472-0

**Published:** 2017-08-08

**Authors:** Jérôme Fernandes, Bruno Bregman, Patrick Combemale, Camille Amaz, Lucie de Léotoing, Alexandre Vainchtock, Anne-Françoise Gaudin

**Affiliations:** 1 Medical Information Department, Groupe OC Santé, Montpellier, France; 2 Bristol-Myers Squibb, Health Economics & Public Health, Bristol-Myers Squibb, 3, rue Joseph Monier, 92500 Rueil-Malmaison, France; 3 Skin Cancer Unit, Medical Oncology Department, Centre Léon Bérard, Lyon, France; 4 Heva, Lyon, France

**Keywords:** Melanoma, Metastatic disease, Hospitalisation, Cost, Immunotherapy

## Abstract

**Background:**

Management of metastatic melanoma is changing rapidly following the introduction of innovative effective therapies, with consequences for the allocation of healthcare resources. The objective of this study was to assess hospitalisation costs of metastatic melanoma in France from 2011 to 2013 from the perspective of the government payer.

**Methods:**

The population studied corresponded to all adults with metastatic melanoma hospitalised in France between 1st January 2011 and 31st December 2013 who required chemotherapy, immunotherapy or radiotherapy due to tumour progression and unresectable Stage III or Stage IV melanoma. Metastatic melanoma was identified by ICD-10 codes documented in the hospital patient discharge records. For each patient, hospital stays were stratified into a pre- or post- progression health state using proxy variables for the RECIST criteria. All healthcare expenditure documented in the French national hospital claims system database and incurred between the index hospitalisation (or change of progression state) and the end of follow-up were analysed. For the principal analysis, valuation of healthcare resource consumption was performed using official national hospitalisation tariffs. Any expensive therapy administered during the stay was documented from a linked database of expensive drugs (FICHCOMP).

**Results:**

Seventy-eight thousand seven hundred fifty hospital stays by 10,337 patients with metastatic melanoma were identified over the three-year study period. Annual per capita costs of hospitalisation were € 5046 in the pre-progression stage and € 19,006 in the post-progression stage. Hospitalisations attributed to adverse drug reactions to chemotherapy or immunotherapy were observed in 27% of patients. Annual per capita costs of these hospitalisations related to adverse drug reactions were € 3762 in the pre-progression stage and € 5523 in the post-progression stage.

**Conclusions:**

Hospitalisation costs related to metastatic melanoma rise substantially as the disease progresses. Treatment strategies which slow down disease progression would be expected to reduce costs of hospitalisation for metastatic melanoma, although they may also entail significant acquisition costs. This will entail organisational changes of resource allocation for the treatment of metastatic melanoma in hospitals.

**Electronic supplementary material:**

The online version of this article (doi:10.1186/s12913-017-2472-0) contains supplementary material, which is available to authorized users.

## Background

Melanoma is the seventh most frequent type of cancer in Europe, with an age-standardised incidence rate of 11.1 cases per 100,000 per year in 2012 [1]. Even though it contributes a relatively small proportion to all skin cancers, melanoma accounts for the majority of skin cancer-related mortality [2]. In France, 1840 deaths were attributed to melanoma in 2012. The relatively high mortality rate associated with melanoma can be explained by late diagnosis of a cancer with a high propensity for metastasis. If detected at an early stage, the tumour can generally be eradicated definitively by surgery, but metastatic disease has a poor prognosis of, with an estimated median survival of 6 months, a one-year survival rate of around 25% and less than 5% of patients surviving for more than 5 years [3–5].

Although the costs of management of metastatic melanoma in individual patients, notably hospitalisation and chemotherapy, may be elevated, the overall contribution of melanoma to hospital expenditure for cancer has, until recently, been modest due to the absence of innovative and therefore costly therapy and to short survival time. A survey of the economic burden of melanoma in France in 2004 [6] estimated the annual hospital costs of melanoma to be € 59 million, of which around one half (€ 27 million) were attributable to metastatic disease.

Many chemotherapeutic agents have been investigated in the treatment of metastatic melanoma over the last 50 years, but overall response and survival rates have consistently been very low [7] and no treatment has been demonstrated to be cost-effective at conventional thresholds [8, 9]. For this reason, unresectable or metastatic melanoma has remained until very recently an area of high unmet medical need. However, over the past 5 years, several new treatments for metastatic melanoma have been introduced which have, for the first time, been shown to provide significant survival benefits [10]. These include small molecule inhibitors of the B-raf/MAP kinase pathway (BRAF inhibitors), such as vemurafenib, dabrafenib and trametinib, and monoclonal antibodies targeting the immune system, such as ipilimumab, directed against CTLA-4, and, more recently, nivolumab and pembrolizumab, directed against the programmed cell death receptor PD-1. As a result, the treatment landscape of metastatic melanoma is changing rapidly.

The introduction of these new innovative and effective therapies for metastatic melanoma is expected to have important repercussions on the cost of care of melanoma. In order to foresee and plan future resource allocation for melanoma care, it is important to have an accurate picture of current patterns of healthcare expenditure on melanoma on the threshold of this major transformation of treatment paradigms. Moreover, Health Technology Assessment bodies have been requesting evaluations of the potential economic impact of new immunotherapies in metastatic melanoma, and the treatment regimens in which they are used, to support decisions on pricing. Since no comprehensive economic evaluation of the management of melanoma in France has been undertaken since 2004, a reappraisal thus seems timely. The present study was undertaken with the objective of assessing the hospitalisation costs of melanoma in France in the metastatic setting over the three-year period from 2011 to 2013, since this is expected to be a major driver of direct medical costs, and is likely to be sensitive to the impact of the introduction of new effective therapies. The primary objective of this study was to estimate the cost per patient-year of hospitalisations related to metastatic melanoma, stratified by pre-progression and post-progression health states. Secondary objectives include estimation of the costs of specific chemotherapies and immunotherapies, the costs of clinical events leading to hospitalisation potentially related to melanoma chemotherapy or immunotherapy.

## Methods

This cohort study (MELISSA) was a retrospective analysis of data on hospitalisations and associated costs extracted from the French national hospital claims system database (PMSI, *Programme de Médicalisation des Systèmes d’Information*) relating to all patients with a first hospitalisation with metastatic melanoma between 1st January 2011 and 31st December 2013. Costs were analysed from the government payer perspective. A brief description of the French health system is provided in Additional file 1.

### Data source

Data were extracted PMSI database. This database contains exhaustive medico-administrative information registered in the patient medical records for all hospitalisations in France in acute care (medicine, surgery or obstetrics), short-term rehabilitation facilities and home care, covering both the public and private sectors. For the present study, stays in psychiatric institutions were excluded from the analysis. In 2013, a total of around 26.5 million individual hospital stays were documented in the PMSI acute care database. Individual patients can be tracked across multiple hospitalisations through a unique anonymous patient identifier, which is conserved until the patient dies. The reasons for hospitalisation are identified for each DRG using the diagnostic codes of the International Classification of Diseases 10th Revision (ICD-10) [11]. These are classified either as principal diagnoses (PD; the condition for which the patient was hospitalised), related diagnoses (RD; any underlying condition which may have been related to the PD, such as a cancer associated with a chemotherapy session) or as significantly associated diagnoses (SAD; comorbidities which may affect the course or cost of hospitalisation). Information on all procedures undergone and treatments provided during hospitalisation is documented by stay at the time of final discharge in the form of a standardised discharge summary (SDS). All medical and surgical procedures are coded according to the French medical classification for clinical procedures (*Classification commune des actes médicaux* [CCAM]) and integrated into a diagnosis-related group (DRG) which is used for classification and valuation of the stay. Demographic data is limited to age, gender and home address postcode. Information on the source of admission and destination of discharge (home, long-term care facility or other hospital) is also available for each stay.

### Study population

The study population comprised all adults (≥18 years at the time of first hospitalisation) with metastatic melanoma hospitalised in France between 1st January 2011 and 31st December 2013 who required chemotherapy, immunotherapy or radiotherapy due to tumour progression and unresectable Stage III or Stage IV melanoma. This population corresponds to patients eligible for immunotherapy with ipilimumab.

In order to align all included patients at the same stage of disease (first hospitalisation for treatment of metastatic disease), the analysis was restricted to patients who had not previously been treated for metastatic melanoma. To this end, a retrospective search of the PMSI database was performed to identify and exclude any patients with a metastatic melanoma-related hospital stay in the 2 years prior to the index hospitalisation using the same decision rules (see below) as those used for inclusion (see below).

Overall, 78,750 hospital stays by 10,337 patients with metastatic melanoma (and no other primary cancer) identified in the SDS were listed in the PMSI database for the 3 years of the study (2011–2013). Of these, 8294 stays (10.5%) were excluded since they were not directly related to melanoma, and 7188 stays (9.1%) related to melanoma were excluded since they had previously received chemotherapy for melanoma and were thus not treatment naïve. The remaining 63,268 stays made by 8862 treatment-naïve patients with metastatic melanoma were the subject matter of the study (Fig. 2).

### Definition of progression stages

For each patient, each hospital stay was stratified according to its progression health state compared with the previous stay using proxy variables in the SDS. Progression was defined as the occurrence of any new metastasis or any treatment change since the previous hospitalisation. If radiotherapy was given at least 2 weeks after initiation of chemotherapy or immunotherapy, this was defined as progression. If the patient received radiotherapy prior to initiation of chemotherapy or immunotherapy, the patient was assigned to the pre-progression state. Patients with cerebral metastases or requiring palliative care at the time of the index hospitalisation were assigned de facto to the post-progression state at the index hospitalisation.

### Identification of hospital stays related to melanoma

(Table 1)

**Table 1:**
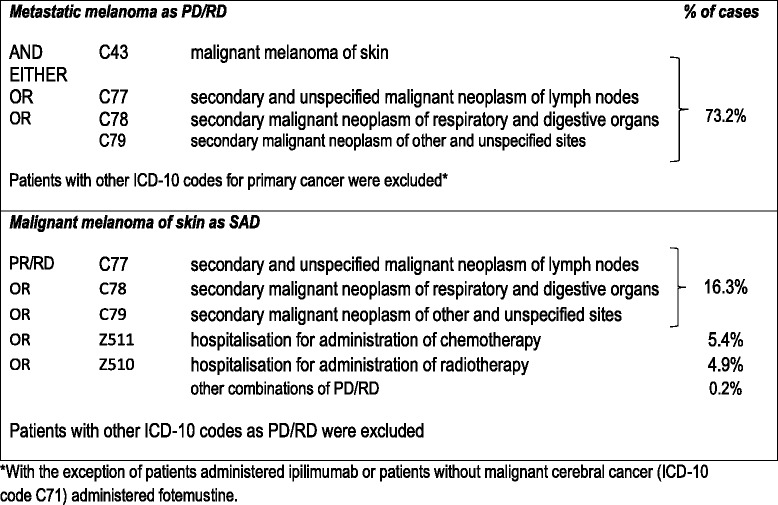
Listing of ICD-10 codes used to assign diagnosis of metastatic melanoma

Proxy markers in the SDS from the hospital stay were defined in order to match this target population as closely as possible. Metastatic melanoma was identified by an ICD-10 code for melanoma (C43: malignant melanoma of skin) as PD, RD or SAD associated with at least one ICD-10 code for metastasis (C77: secondary and unspecified malignant neoplasm of lymph nodes; C78: secondary malignant neoplasm of respiratory and digestive organs or C79: secondary malignant neoplasm of other and unspecified sites). Stays with another ICD-10 code for primary cancer in the SDS were excluded, since it was not possible to determine whether the metastasis was due to melanoma or to the other primary cancer, with two exceptions. These were administration of ipilimumab (since this treatment is only indicated in melanoma) or administration of fotemustine to patients without malignant cerebral cancer (ICD-10 code C71), since this is the only other indication apart from melanoma for which fotemustine is approved.

All stays for which the PD/RD was identified with the ICD-10 code C43 (malignant melanoma of skin) were retained. For stays in which malignant melanoma of skin was documented as a SAD, decision rules were established to assign hospital stays to either ‘directly related’ or ‘not related’ to malignant melanoma of the skin according to the PD/RD codes for the stays. These decision rules were established a priori by a clinician specialised in skin cancer and a coding physician expert on the PMSI for a possible relationship with malignant melanoma of skin. Only stays which were considered to have a direct relationship with malignant melanoma of skin were retained. Three possible situations arose. Firstly, when the PD/RD was clearly associated with malignant melanoma of skin, the stay was retained. This was the case, for example, for stays where the ICD-10 codes for the PD/RD related to metastatic disease (C77, C78, C79) or to hospitalisation for administration of chemotherapy session (Z511). Secondly, when the PD/RD was clearly associated with a pathology other than malignant melanoma, the stay was excluded (for example, a patient hospitalised following a road accident). Finally, when the association with malignant melanoma of skin was not obvious, a conservative approach was adopted and such stays were excluded.

### Data sources

Using the patient unique identifier number, all hospital stays from the first documented during the study period until the patient dies or until the 31st December 2013 (whichever came first) were extracted for costing. Stays considered unrelated to melanoma were excluded using the same rules as those applied for patient eligibility. Each stay related to melanoma was assigned to a pre- or post- progression state as specified above. Stays related to administration of chemotherapy or immunotherapy were identified from the DRG codes assigned to ‘*Sessions of chemotherapy for tumour* (*ambulatory*)’ and ‘*Chemotherapy for tumour* (*full hospitalisation*)’ (codes 28Z07Z and 17 M06*). The FICHCOMP database was used to identify any stay at which fotemustine or ipilimumab had been administered. In addition, stays presumed to be related to treatment of adverse drug reactions to melanoma treatment were extracted.

### Data collection

All documented healthcare resource consumption in the PMSI database was identified for the period between the index hospitalisation (or change of progression state) and the end of follow-up. The end of follow-up was defined as the end of the observation period (31st December 2013), death or loss to health insurance of the patient, or change of health state due to disease progression. Any acquisition costs of fotemustine or ipilimumab during this period were identified from the FICHCOMP database.

Healthcare resources documented in the PMSI database include costs of radiotherapy, acquisition costs for standard chemotherapy (acquisition costs for immunotherapy and innovative chemotherapy are documented separately in the FICHCOMP database) and administration costs for all chemotherapy and immunotherapy. They also include all diagnostic tests performed during hospital stays or day hospitalisations (for example, PET, MRI, CT), all medical and paramedical care provided in hospital including palliative care, intensive care and psychosocial support.

Healthcare resources not documented in the PMSI database include any consultations in community medicine (for example, primary care, community-based specialists, psychological support, nursing), as well as outpatient hospital consultations, medications prescribed outside the hospital (and notably BRAF inhibitors), laboratory and other diagnostic tests (for example, echography, CT scans). In patients followed in large university hospitals, most diagnostic tests and consultations with other physicians are generally provided during a comprehensive day hospitalisation, whereas for patients followed in small local hospitals, these services are generally provided in the community. Furthermore, hospice care is not covered by the PMSI database.

### Determination of mortality

Mortality was documented through the PMSI database, where all in-hospital deaths are identified although cause of death is not reported. Mortality documented in the PMSI database thus does not fully reflect death from metastatic melanoma for two reasons, firstly because deaths occurring outside hospital are not accounted for and secondly because in-hospital deaths due to causes other than melanoma such as accidents may artificially inflate mortality rates. These sources of imprecision are expected to be relatively minor, since the vast majority of patients with cancer in France die in hospital [12] and most patients with metastatic melanoma die from it [13]. In order to take into account these sources of imprecision, a correction was made using data from the national deaths registry in which all deaths in France are entered, together with the cause of death. This correction was based on a comparison between the number of deaths due to melanoma in 2011 documented in the national deaths registry (1732 deaths) and the corresponding number of all-cause deaths in patients with metastatic melanoma documented in the PMSI database over the same period (1368 deaths). This indicates that 79% of deaths occur in hospital and 21% elsewhere. For each year of follow-up, the number of non-hospital deaths was thus estimated as 0.21/0.79 × the number of in-hospital deaths.

### Outcomes

As specified in the study protocol, the costs per patient-year of all hospitalisations related to metastatic melanoma were determined, both overall and for hospitalisations at which chemotherapy or immunotherapy was administered. Such hospitalisations were identified through documentation in the FICHCOMP database for fotemustine or ipilimumab and through documentation of the ICD-10 code for administration of antineoplastic chemotherapy (Z511) in the SDS for other treatments. These hospitalisations included both programmed visits, for example for chemotherapy or radiotherapy sessions, and unprogrammed visits, for example for management of an adverse drug reaction.

The costs per patient-year of all hospitalisations related to clinical events presumably related to melanoma immunotherapy (ipilimumab) and BRAF targeted treatments were also determined. Since the cause of clinical events is not explicitly defined in the SDS, this could not be directly attributed to melanoma treatment. For this reason, a list of frequently associated adverse drug reactions was established a priori by the Steering Committee of the study. These events included infections, myalgia/pain, skin reactions, fatigue, diarrhoea, nausea/vomiting, colitis, dyspnoea, anaemia, thrombocytopenia, neutropenia, decreased blood corticotrophin, glomerulonephritis, skin carcinoma and neuropathies (sensory/motor). All additional stays of each patient for which these ADRs were documented in the SDS as a PD/RD (irrespective of whether metastatic melanoma was documented as an SAD) were extracted from the PMSI database and the related cost determined. The costs per patient-year of all hospitalisations involving palliative care were also determined. These hospitalisations can be identified through the relevant ICD-10 code (Z51.5) in the SDS.

### Valuation

Healthcare resource consumption costs were determined for each progression state and for each patient using three different costing conventions. The first of these used official French tariffs which are established by the national health insurance system for accounting and resource allocation purposes. In our study, the total per capita cost accrued was calculated from the DRG-specific unit tariffs attributed to each stay, together with the cost of any expensive chemotherapy or immunotherapy administered during the stay that was documented in the FICHCOMP database. The DRG-specific unit cost was adjusted for the actual length of the hospital stay by adding or subtracting daily hospitalisation rates. The second valuation method used the unit costs determined in the *Études Nationales de Coûts à Méthodologie Commune* (ENCC). This is a bottom-up costing study in a representative sample of all French hospitals designed to evaluate the real DRG-based aggregate costs of hospitalisation. These cost determinations are important for the national health insurance system to align national tariffs with real costs. The mean ENCC costs include the cost of expensive chemotherapy. The third valuation method used the DRG-specific unit ENCC costs adjusted for the use of expensive chemotherapy and immunotherapy. For each stay, the cost of expensive drugs determined in the ENCC was subtracted from the total cost of the stay and replaced by the actual cost of reimbursed drugs as specified in the FICHCOMP database administered during the individual hospital stay, on a patient-by-patient basis. All costs are presented as inflation-adjusted 2017 Euros.

### Data analysis

**Fig. 1:**
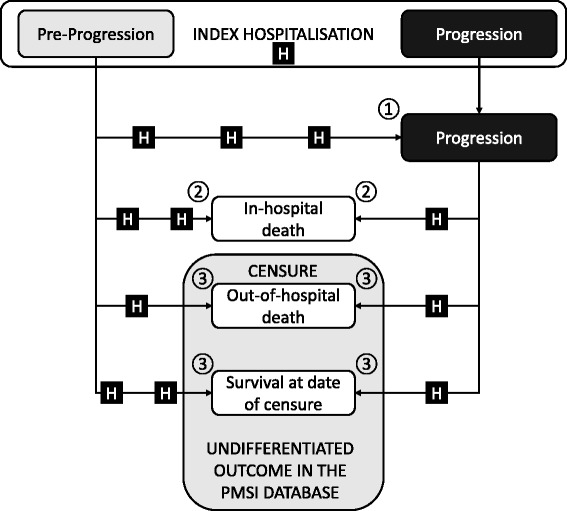
Patient trajectories in the PMSI database. Patients are identified in the PMSI database when they undergo a hospitalisation fulfilling the eligibility criteria for metastatic melanoma. This is the index hospitalisation. At this time, the patient is attributed to the pre-progression stage or the post-progression stage. The patient is followed until one of three endpoints is reached: 1. the patient changes progression stage (only changes from pre-progression to post-progression are possible); 2. the patient dies in hospital, which is recorded in the database; 3. censure at the end of the follow-up period (31st December 2013). In the last case it is not known whether the patient has died at home or is still alive, since out-of-hospital deaths are not recorded in the database. Between the index hospitalisation and any one of these endpoints, the patient may undergo one or more hospitalisations (H) which are accounted separately for the pre-progression stage and the post-progression stages

The possible trajectories of individually patients that can be followed in the PMSI database are illustrated in Fig. 1. The individual follow-up duration in days of each patient per progression health stage was determined. In the pre-progression stage, the duration of follow-up time was defined for each patient as the time between the date of index hospitalisation and the date of progression, the date of death or the date of censuring (31st December 2013). In the post-progression stage, the duration of follow-up time was defined for each patient as the time between the date of progression and the date of death or the date of censuring. For patients who progressed, the date of the hospitalisation at which the criteria for progression were met was taken as the date of progression. Certain patients were considered to be in the post-progression stage at the time of the index hospitalisation (see above).

Since it was not possible to know whether individual patients who were censured had died or were still alive, an adjustment was made for the probability of dying outside hospital at the population level. An unadjusted and an adjusted follow-up duration was determined and used to calculate the per capita annual costs. For the unadjusted follow-up duration (base case), the individual follow-up durations in days for each patient in progression each stage were summed to generate a cumulative follow-up duration and this was then divided by the cumulative costs generated by the same cohort of patients to generate the per capita cost:

$$ Annal\  per\  capita\  cost=\frac{Cumulative\  cost s\  per\  progression\  stage\ \left(\mathit{{\textsf{C}\hspace{-1.7ex}{=}}}\right)}{Cumulative\  time\  in\  progression\  stage\ (days)}\times 365 $$

In the survival-adjusted analysis, the individual follow-up durations in days for each patient in progression each stage were summed separately for patients who died in hospital, for patients who progressed and for patients who were censured. For the latter group, the cumulative follow-up duration was then truncated to take into account the shorter actual follow-up duration of patients who died outside hospital. In order to do this, the number of patients estimated to have died outside hospital for each of the three calendar years (2011, 2012 and 2013) was calculated as described above (determination of mortality). As a conservative hypothesis, these deaths were assumed to have occurred on the 1st January of the given calendar year. For the number of patients assumed to have died in each calendar year, the duration of the remaining follow-up time from the 1st January of the year of death to the end of the follow-up period (date of censure: 31st December 2013) was determined and subtracted from the unadjusted cumulative follow-up duration as follows:

$$ Adjusted\  cumulativ e\  follow- up\  duration\ (days)= Unadjusted\  cumulativ\  follow- up\  duration(days)-{\left[ Number\  of\  estimated\  deaths\  in\ 2011\right]}^{\ast }365\times 3-{\left[ Number\  of\  estimated\  deaths\  in\ 2012\right]}^{\ast }365\times 2-{\left[ Number\  of\  estimated\  deaths\  in\ 2013\right]}^{\ast }365\times 1 $$

The presentation of the data is descriptive and no a priori statistical hypotheses were tested. Categorical variables are described as frequency counts and proportions with their 95% confidence intervals (95% CI). Continuous variables are described as mean values with standard deviation (or 95% CI) or as median values [range]. All analyses were performed using SAS™ software Version 16.4 (Cary, USA).

## Results

### Patient cohort

**Fig. 2:**
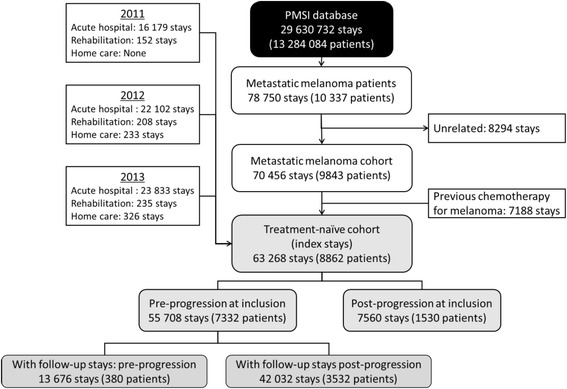
Description of selection of hospital stays. PMSI: Programme de Médicalisation des Systèmes d’Information (French national hospital database)

Overall, 78,750 hospital stays by 10,337 patients with metastatic melanoma (and no other primary cancer) identified in the SDS were identified in the PMSI database for the 3 years of the study (2011–2013). Of these, 8294 stays (10.5%) were excluded since they were not directly related to melanoma. Of the remaining 70,456 stays, 63,268 (89.8%) were made by 8862 patients who had not received chemotherapy for melanoma in the previous 2 years, and these formed the study population (Fig. 2). The overall mean number of melanoma-related stays per patient was 7.14. The annual number of stays increased somewhat over the 3 years of the study. The vast majority of the stays were in acute stays in general hospitals (96.6%). A total of 26,843 stays (42.4%) concerned 7332 patients who were at the pre-progression stage and the remaining stays concerned 5062 patients at the post-progression stage. Many individual patients (*N* = 3532; 39.9%) underwent stays during both pre- and post- progression stages and are thus represented in both subgroups. The mean number of stays per patient was 3.66 for the pre-progression subgroup and 7.20 for the post-progression subgroup. The mean duration (± SD) of the hospital stays was 2.7 ± 10.6 days for stays during the pre-progression stage and 3.1 ± 9.4 days for stays during the post-progression stage. Men were slightly over-represented in the study cohort and the mean age of the enrolled patients was 63.7 years (Table 2).

**Table 2:** Patient characteristics

	Pre-progression(*N* = 7332)	Post-progression(*N* = 5062)	Total(*N* = 8862)
Gender			
Men	3894 (53.1%)	2829 (55.9%)	4764 (53.8%)
Women	3438 (46.9%)	2233 (44.1%)	4098 (46.2%)
Age (years)			
Mean ± SD	63.4 ± 15.9	64.2 ± 15.7	63.7 ± 15.9
Median (Min. – Max.)	65 (18–103)	66 (18–99)	65 (18–103)
Initial progression marker			
Cerebral metastases^a^		1348 (26.6%)	
New metastases in other sites		2027 (40.0%)	
Palliative care		1058 (20.9%)	
With cerebral metastases		300 (5.9%)	
Without cerebral metastases		758 (15.0%)	
Treatment change		629 (12.4%)	

^a^Without delivery of palliative care

Overall, 3479 patients (39.3%) died in hospital. The crude mortality rate was tenfold higher in patients at the post-progression stage (3049, 60%) than at the pre-progression stage (430, 6%). 925 patients were estimated to have died elsewhere. A total of 1056 patients received palliative care (11.9%); by definition, all patients receiving palliative care were assigned to the post-progression stage.

For the 5062 patients with documented disease progression, the initial progression marker was detection of new metastases in 3675 cases (72.6%), delivery of palliative care in 1058 cases (20.9%) and need for a change in treatment in 629 patients (12.4%) (Table 2).

### Total costs

Applying the official French hospital tariffs, the annual per capita costs of hospitalisation for metastatic melanoma were € 5046 for patients in the pre-progression stage and € 19,006 for those in the post-progression stage (Table 3). Very similar cost values were obtained when using mean unit real costs derived from the ENCC surveys instead of the national tariffs. Adjustment of the ENCC unit costs to take into account the reimbursable cost of any expensive chemotherapy actually delivered led to a small decrease of per capita costs of around 5% for patients in the pre-progression stage and a more substantial increase of around 15% for those in the post-progression stage (Table 3).

**Table 3:** Annual per capita costs of hospitalisation for metastatic melanoma

	Patients	Stays	Annual per capita cost		
			National tariff	ENCC	Adjusted ENCC
Pre-progression	7332	26,843			
Base case			€ 5001	€ 5027	€ 4803
Survival-corrected			€ 5178	€ 5205	€ 4973
Post-progression	5062	36,425			
Base case			€ 18,839	€ 15,667	€ 18,155
Survival-corrected			€ 33,537	€ 27,890	€ 32,320

ENCC *Études Nationales de Coûts à Méthodologie Commune*

When costs were adjusted for survival, on the grounds that certain patients would die outside hospital between the time of their last hospitalisation and the end of the study period (Table 3), the per capita costs increased marginally for patients in the pre-progression stage (from € 5046 to € 5224 using national tariffs) and much more sizeably for those in the post-progression stage (from € 19,006 to € 33,835).

### Costs of chemotherapy or immunotherapy

Annual per capita costs were assessed for hospitalisations related to metastatic melanoma with administration of ipilimumab or fotemustine (both specifically identified in the FICHCOMP database), or of all other chemotherapeutic agents. These costs are presented in Table 4.

**Table 4:** Annual per capita costs of chemotherapy and immunotherapy for metastatic melanoma

	Patients	Stays	Annual per capita cost		
			National tariff	ENCC	Adjusted ENCC
*Ipilimumab*					
Pre-progression	55	165	€ 64,017	€ 3949	€ 63,895
Acquisition	(0.8%)	(0.6%)	€ 62,215	NA	€ 62,215
Administration			€ 1802	NA	€ 1680
Post-progression	348	949	€ 43,735	€ 2529	€ 43,657
Acquisition	(6.9%)	(2.6%)	€ 42,530	NA	€ 42,530
Administration			€ 1205	NA	€ 1127
*Fotemustine*					
Pre-progression	139	541	€ 5194	€ 5937	€ 5044
Acquisition	(1.9%)	(2.0%)	€ 2372	NA	€ 2372
Administration			€ 2822	NA	€ 2672
Post-progression	969	3192	€ 3432	€ 3603	€ 3337
Acquisition	(19.1%)	(8.7%)	€ 1696	NA	€ 1696
Administration			€ 1736	NA	€ 1641
*Other chemotherapies* ^a^					
Pre-progression	1475(20.1%)	4845(18.0%)	€ 3378	€ 6205	€ 3252
Post-progression	2052(40.5%)	10,214(28.0%)	€ 2736	€ 5292	€ 2601

*NA* not available

^a^Since other chemotherapies are not included in the FICHCOMP list, drug acquisition and administration costs are consolidated for all three valuation methods. ENCC: *Études Nationales de Coûts à Méthodologie Commune*

Of the 7332 patients in the pre-progression stage, 55 (0.8%) received ipilimumab at 165 individual stays. During this stage, the per capita cost of ipilimumab using national tariffs was € 64,585. During the post-progression stage, 348 patients (6.9%) received ipilimumab at a mean per capita cost of € 44,124. In both stages, when the ENCC unit costs were used for the valuation, the costs of therapy were estimated to be sixteen-fold lower. When the ENCC unit cost was adjusted for the real reimbursable cost of the treatment actually delivered, a per capita cost close to that estimated from national tariffs was obtained (Table 4). Both using national tariffs and adjusted ENCC unit costs, acquisition costs far outweighed administration costs, accounting for >95% of the total cost of immunotherapy. If costs were adjusted for survival, the costs of ipilimumab were increased by ≤10%, according to the valuation convention used (data not shown).

In the case of fotemustine, 139 patients (1.9%) received this treatment in the pre-progression stage and 969 patients (19.1%) during the post-progression stage. Per capita costs using national tariffs were € 5240 and € 3463 respectively. In this case, cost determined using ENCC unit costs were somewhat higher than costs derived from national tariffs, by a factor of around 10% (Table 4). Again, after adjustment for the treatments actually received, the two methods generated rather similar cost estimates. For fotemustine, drug acquisition and administration costs were within the same order of magnitude.

For other chemotherapeutic agents which do not figure on the list of expensive treatments eligible for supplementary funding, drug acquisition costs are integrated into the unit cost for the DRG. It was therefore not possible to distinguish between acquisition and administration costs for these agents. Such treatments were delivered to 1475 patients (20.1%) at the pre-progression stage and 2052 patients (40.5%) at the post-progression stage. The per capita cost according to national tariffs was € 3408 at the pre-progression stage and € 2760 at the post-progression stage. Using ENCC costs as the basis for the valuation, per capita costs were around twice as high, although this difference was abolished after adjustment for the actual expensive drugs delivered.

### Costs related to adverse drug reactions to chemotherapeutic or immunotherapeutic agents

Overall, 5817 hospital stays where identified in 2379 patients that were attributed to the management of adverse drug reactions to immunotherapy of chemotherapy. These stays were documented in 1223 patients (16.7%) during the pre-progression stage and in 1354 patients (26.7%) during the post-progression stage (Table 4). The annual per capita costs of these stays were € 3762 in the pre-progression stage and € 5523 in the post-progression stage (national tariffs). These costs were essentially similar whatever the valuation convention used. When corrected for out of hospital mortality, the per capita cost accrued during the post-progression period increased from € 4015 to € 8658 (Table 5). The most frequent of these adverse drug reactions were skin carcinoma, infections and anaemia, and the most costly to manage were infections, neutropenia and glomerulonephritis (Table 5).

**Table 5:** Costs of management of adverse events attributed to chemotherapy

	National tariff	ENCC	Adjusted ENCC
*All ADRs – Annual* per capita *costs*			
Pre-progression (*N* = 1223 patients)			
Base case	€ 3729	€ 3947	€ 3915
Survival-corrected	€ 3979	€ 4212	€ 4177
Post-progression (*N* = 1354 patients)			
Base case	€ 5474	€ 5452	€ 5631
Survival-corrected	€ 8582	€ 8546	€ 8827
*Individual ADRs* (*both progression stages combined*) – Mean cost per stay with ADR^a^			
Infections (*N* = 1090 stays)	€ 3936 ± 4328	€ 4184 ± 4301	€ 4214 ± 4451
Neutropenia (*N* = 225 stays)	€ 3806 ± 3920	€ 4115 ± 3877	€ 3946 ± 3988
Glomerulonephritis (*N* = 41 stays)	€ 3456 ± 3552	€ 3713 ± 4099	€ 3698 ± 4017
Myalgia/pain (*N* = 309 stays)	€ 3032 ± 4609	€ 2580 ± 2213	€ 2940 ± 4468
Colitis (*N* = 93 stays)	€ 2942 ± 2114	€ 3107 ± 2078	€ 3105 ± 2130
Diarrhoea (*N* = 40 stays)	€ 2747 ± 3036	€ 3043 ± 4158	€ 3104 ± 4376
Decreased blood ACTH (*N* = 34 stays)	€ 2607 ± 2725	€ 2410 ± 1907	€ 2454 ± 2143
Fatigue (*N* = 268 stays)	€ 2567 ± 2817	€ 2405 ± 1771	€ 2541 ± 2549
Skin reactions (*N* = 137 stays)	€ 2333 ± 2544	€ 2278 ± 1647	€ 2398 ± 2514
Dyspnoea (*N* = 56 stays)	€ 1875 ± 1508	€ 1896 ± 1487	€ 1918 ± 1512
Anaemia (*N* = 660 stays)	€ 1645 ± 2510	€ 1726 ± 1554	€ 1791 ± 2370
Nausea/vomiting (*N* = 42 stays)	€ 1302 ± 703	€ 1539 ± 796	€ 1532 ± 792
Thrombocytopenia (*N* = 208 stays)	€ 1252 ± 1432	€ 1929 ± 2159	€ 1392 ± 1330
Basocellular carcinoma (*N* = 2599 stays)	€ 1129 ± 2671	€ 1073 ± 1922	€ 1116 ± 2553
Neuropathies (*N* = 15 stays)	€ 691 ± 597	€ 1489 ± 610	€ 808 ± 586

^a^Mean costs are calculated for the total number of stays related to each individual ADR and are presented with their standard deviations. ENCC: *Études Nationales de Coûts à Méthodologie Commune*

### Costs related to hospitalisations with palliative care

A total of 1883 patients (37.2%) in the post-progression stage underwent hospitalisations with palliative care. The annual per capita cost of these stays was € 20,107 (national tariffs), € 21,101 (ENCC unit costs) or € 20,070 (adjusted ENCC unit costs).

## Discussion

This study provides the first estimation of hospital costs specifically associated with metastatic melanoma in France since the introduction of effective targeted chemotherapy and immunotherapies. The primary objective of this study was to determine the annual per capita costs of hospitalisation for metastatic melanoma in treatment-naïve patients in France, according to the progression stage. These costs rose substantially as the disease progressed from hospitalisation for metastatic melanoma were € 5046 in the pre-progression stage to € 19,006 in the post-progression stage. Given the number of patients hospitalised over the 3 years of the study, this represents a total cost to the health service of € 130 million. Expressed as yearly expenditure, this represents an increase of expenditure on metastatic melanoma of 60% since the last evaluation performed in 2004. Nevertheless, this still remains a small proportion of the total direct healthcare expenditure on cancer in France, which amounted to € 7000 million in 2009 [14].

The annual per capita costs of treatment were also determined. Ipilimumab (an anti- CTLA-4 immunotherapy for advanced melanoma) and fotemustine (a chemotherapy for melanoma with cerebral metastases) are the only two drugs that could be individualised, as these are the only two treatments of advanced melanoma documented in the FICHCOMP database. Ipilimumab was only used in 402 patients (<5%), principally in the post-progression stage (348 patients). The limited use of ipilimumab in this study reflects the fact that it was only available through a named-patient early access scheme prior to its commercialisation in France in March 2013 for half the period of the study. Its use in post-progression patient reflects the fact that it was only licensed as a second-line treatment for advanced/metastatic melanoma at the time of the study. The annual per capita treatment cost of ipilimumab was substantial (€ 64,585 in the pre-progression stage and € 44,124 in the post-progression stage), reflecting its high acquisition cost. The overall contribution of ipilimumab to total costs of metastatic melanoma is likely to have increased since the time of the study, leading to a consequent inflation of total costs, as a result of more widespread use of this treatment and its availability as a first-line treatment for advanced/metastatic melanoma since 2014. With respect to fotemustine, this is only used in patients who develop cerebral metastases. The annual per capita treatment cost of fotemustine was over tenfold lower than that of ipilimumab, € 5240 in the pre-progression stage and € 3463 in the post-progression stage. This cost was comprised in roughly equal parts of the acquisition and the administration costs, perhaps reflecting the need for relatively frequent administration under medical supervision.

The impact of the introduction of immunotherapies such as ipilimumab on the direct medical costs of management of metastatic melanoma would be expected to be two-fold. Firstly, since these agents reduce the risk of progression [15], and since progression is associated with a nearly four-fold increase in hospitalisation costs, their use would be expected to decrease these costs. On the other hand, this gain would be to some extent offset by the significant acquisition cost of ipilimumab. It should be noted that since the period of this study, PD-1 inhibitors (nivolumab and pembrolizumab) have been introduced in France for the immunotherapy of metastatic melanoma. These agents are more effective than ipilimumab in reducing the probability of progression [16, 17] and thus would be expected to offer a greater reduction in hospitalisation costs. On the other hand, PD-1 inhibitors are potentially used for longer periods (until progression) than ipilimumab (a single three-month course), which may generate higher acquisition costs. In addition, the use of combinations of different immunotherapeutic agents may produce further reductions in post-progression hospitalisation costs, but also generate higher cost related to management of adverse events, which would be expected to be more frequent [18]. It would be of interest to perform a new cost analysis to evaluate the impact of these new therapies.

Concerning hospitalisations attributed to adverse drug reactions to chemotherapeutic or immunotherapeutic agents, these were observed in around 27% of patients overall, and more frequently during the post-progression stage. The annual per capita costs of these events corresponded to € 3762 in the pre-progression stage and to € 5523 in the post-progression stage. However, it should be noted that costs per stay with ADR were highly variable depending on the type of adverse event reaction considered, being most expensive for infections and least expensive for neuropathies. The actual costs of managing adverse drug reactions related to immunotherapy or chemotherapy for melanoma determined in our study are much lower than the costs estimated in a recent literature review of the costs of in-hospital management of toxicities associated with treating metastatic melanoma in eight countries, including France [19], which ranged from € 1416/event for cutaneous squamous cell carcinoma to € 6913 for hepatotoxicity. Similarly high costs associated with adverse drug reactions to metastatic melanoma treatments have also been reported in an analysis of data from a North American prescription claims database [20].

In this study, we compared cost valuation using national tariffs to valuation using mean real-world costs obtained from the ENCC surveys. With respect to total costs, the two valuation methods provided very similar estimates, suggesting that, for the treatment of metastatic melanoma, national tariffs accurately reflect the actual cost of in-hospital management. However, when treatment costs were considered, large discrepancies between the two methods were observed. In the case of patients treated with ipilimumab, cost estimates based on national tariffs and including ipilimumab acquisition costs from the FICHCOMP database were twenty-fold higher than the cost estimates based on the ENCC mean unit costs. This can be explained by the fact that the ENCC costs are generated from patients treated with a range of cancer therapies with different acquisition costs, such that the cost of ipilimumab is diluted by that of less expensive drugs. In contrast, in patients with other chemotherapies that do not figure on the FICHCOMP list (and are therefore in principle inexpensive) the costs estimated from ENCC mean unit costs were nearly twice as high as those estimated from national tariffs. In this case, the ENCC mean unit costs are inflated by the acquisition costs of recent innovative therapies commanding a premium price. In consequence, the best way to estimate the real costs of cancer therapies may be to adjust the ENCC mean unit cost for the cost of the individual treatments received by each patient as documented in the FICHCOMP database. In our study, performing such an adjustment abolished nearly all the difference between costs estimates bases on national tariffs and those based on the ENCC mean unit costs.

Around one-quarter of patients with post-progression metastatic melanoma underwent hospitalisation including palliative care, and this carried a high annual per capita cost (€ 20,107). It is noteworthy that only a minority of patients who finally died in hospital of metastatic melanoma received any palliative care.

Data for the study were obtained from the PMSI database which contains exhaustive information on in-hospital healthcare consumption for all beneficiaries of the principal public health insurance schemes in the country, covering around 97% of the population. For this reason, we are confident that essentially all stays related to melanoma will have been captured in our analysis. The costs obtained in the valuation using national tariffs are relevant as these correspond to the tariffs used for resource allocation in the French health system. Nonetheless, the PMSI database does have several limitations from the perspective of economic analyses. Firstly, it is not possible to be sure that all resource use during hospitalisation was effectively for the treatment of metastatic melanoma or that all hospitalisations in which melanoma was documented as a SAD in the SDS were related to melanoma. We attempted to address this latter uncertainty by screening the PD codes to exclude clearly unrelated hospitalisations, and this led to the exclusion of around 10% of stays. Secondly, the cause of death is not documented in the SDS and out-of-hospital deaths are not documented at all. We assumed that all patients who died whilst hospitalised for metastatic melanoma died as a result of their cancer, but this may well not always be the case, leading to an over-estimation of mortality. We also assumed that the proportion of out-of-hospital deaths to in-hospital deaths was identical for metastatic melanoma as for all-cause mortality in France in order to correct our cost estimates for survival. This correction had a major impact, leading to an increase in total cost of around 80%. For this reason, the uncertainty over the true mortality rate for metastatic melanoma limits the precision of the survival-corrected cost estimate. The number of deaths estimated during the course of the study (3479 in-hospital deaths and 925 out-of-hospital deaths over 3 years) is somewhat lower than the total number of melanoma-related deaths in France in 2012 documented in the EUROCAN registry (1840 deaths) [1]. Thirdly, proxy markers based on the ICD-10 codes listed in the SDS were used to define the study population. Using such proxies, it is not possible to match exactly the target population (patients treated for advanced (unresectable or metastatic) melanoma in adults.) or the progression criteria used in clinical trials, such as RECIST [21], and this may result in some imprecision in the cost estimates. In addition, we have not differentiated between unresectable Stage III cancer and Stage IV cancer in our analysis, as this is not possible using DRG codes, and these groups may bear very different treatment costs. Fourthly, in the analysis of costs attributed to adverse drug reactions to chemotherapy, the relationship of such events to the chemotherapy is not specified in the SDS and cannot be ascertained independently. For this reason, all hospitalisations for one of a list of pre-specified clinical events corresponding to a characterised chemotherapy adverse drug reaction was considered to be treatment-related. Nonetheless, it is possible that not all infections requiring hospitalisations, for example, were in reality attributable to treatment of metastatic melanoma. Fifthly, costs accrued in community and outpatient care are not captured in the PMSI database and are thus not considered in our analysis. In particular, the B-RAF inhibitors vemurafenib and dabrafenib are administered orally and hospitalisation is not required for their use. The acquisition costs of these drugs, which will be substantial, has not been included in our analysis. In addition, prior use of these agents will not have been documented in the PMSI database and thus certain patients that we have considered as treatment-naïve may in reality have previous experience with B-RAF inhibitors. In this respect, it would be interesting to replicate this analysis in the SNIIRAM claims database, which has recently become more readily available for medico-economic studies and covers all reimbursed healthcare resource consumption by all national health insurance beneficiaries in France both in hospital and in community medicine. Finally, a lag time is needed in order to consolidate all expenditures in the PMSI database, and it is thus not possible to measure health expenditure, and changes in expenditure, in real time. In practice, analyses of the database tend to be performed 1 year, if not more, after the expenditure was actually outlaid.

## Conclusions

The findings of this study will be useful as an updated reference for planning resource allocation for the care of patients with metastatic melanoma following the introduction of effective immunotherapies. The observation that hospitalisation costs related to metastatic melanoma rise substantially as the disease progresses suggests that innovative treatment strategies, which have been shown to slow down disease progression, are likely to make a significant impact on the costs of hospitalisation and palliative care which, together with their acquisition costs, will entail organisational changes of resource allocation for the treatment of metastatic melanoma in hospitals.

## Additional file

Additional file 1: Brief description of the French health system. (PDF 401 kb)
