# Supplementary material for: Hospitalisation costs of metastatic melanoma in France; the MELISSA study (MELanoma In hoSpital coSts Assessment)
Source: BMC Health Serv Res. 2017 Aug 8;17:542. doi: 10.1186/s12913-017-2472-0 (PMC5549336; doi:10.1186/s12913-017-2472-0)
Supplement: Additional file 1: — Brief description of the French health system. (PDF 401 kb) [file 12913_2017_2472_MOESM1_ESM.pdf]

Additional file 1: Brief description of the French health system

The French health system is a mixed public/private system, both in terms of the point of care provision and reimbursement. With respect to point of care, healthcare provision is provided in public or private hospitals, or by community physicians. Most care provision is entirely funded by national (public) health insurance, at fixed tariffs set by the Health Ministry. However, specialist physicians exercising totally or partially in the private sector can set prices to the user freely without restriction.

With respect to reimbursement, a variable proportion of cost for each health service (ranging from 0% for non-reimbursed medications to 100% for hospitalisations) is borne by national health insurance and generally paid directly to the provider without the user having to outlay any money. The remaining cost is charged to the user, who may contract a private health insurance to cover these costs. For salaried workers, the employer has a legal obligation to offer such private health insurance to their staff. For care provided in the private sector, national health insurance reimburses an identical cost to that reimbursed in the public sector, the additional cost being borne by the user. In the case of a restricted list of severe chronic diseases established by the Health Ministry, the cost of all care provision related to this disease is fully covered by national health insurance. Cancer is one of these listed diseases and thus all cancer care is provided without the need for any outlay of money by the user.

With respect to funding in the public sector, hospitals are funded on the basis of the number of interventions performed, each intervention being associated with a unit tariff set by the Health Ministry and revised annually. An individual tariff is set for each type of hospital visit on the basis of a diagnosis-related group (DRG) code and represents an aggregate of all care services expected to be used during the stay (for example, hours of nursing time, physician consultations, operating theatre use, radiotherapy, medication prescription, paraclinical examinations or laboratory tests), each service being assigned a standard unit cost. Nonetheless, each hospital stay is different and actual resource utilisation may differ from that envisaged in the 'model' stay used to determine the tariff. For this reason, actual resource utilisation is monitored annually in a sample of 1/20 of public and private hospitals and used to generate a mean actual cost of the intervention which can be used by the Health Ministry to adjust and adapt the standard tariffs if necessary. These annual surveys are published as the *Études Nationales de Coûts à Méthodologie Commune* (ENCC).

Over the last two decades, a number of innovative but expensive medications have been introduced for hospital use and their use may distort the cost of hospital stays with respect to the standard DRG-based tariffs. In order to ensure that use of these drugs is not censored by hospital administrators for economic reasons and that access to them is equitable between different hospitals, these are funded separately from the standard tariff. A list of these medications is maintained by the Health Ministry and many recent cancer treatments are on

this list of medications. In the public sector, these medications are paid for directly by national health insurance, on an as needed basis, and all use is documented in a specific database (FICHCOMP) for monitoring purposes. In the private sector, the cost of these medications over and above the DRG-related tariff is covered by health insurance.
